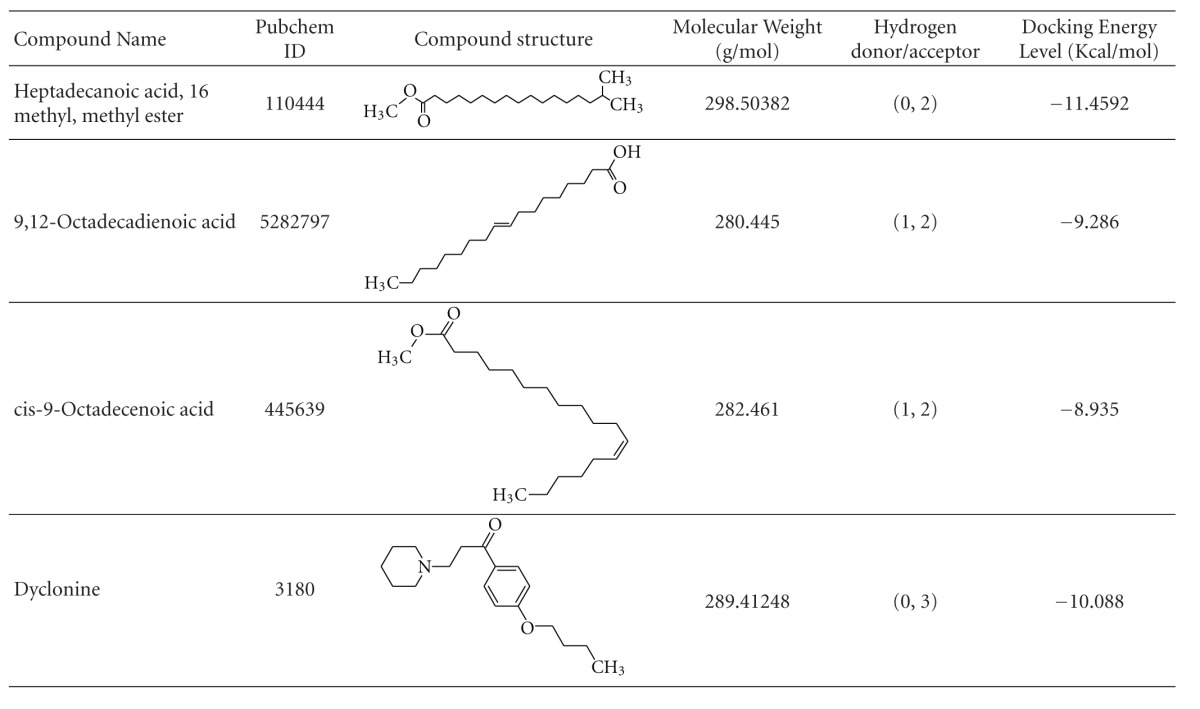# *In Silico* Studies on Fungal Metabolite against Skin Cancer Protein (4,5-Diarylisoxazole HSP90 Chaperone)

**DOI:** 10.5402/2012/626214

**Published:** 2012-09-06

**Authors:** Saravanakumar Kandasamy, Sunil Kumar Sahu, Kathiresan Kandasamy

**Affiliations:** Centre of Advanced Study in Marine Biology, Faculty of Marine Sciences, Annamalai University, Parangipettai 608 502, India

## Abstract

This work was to find out the dominant secondary metabolites derived from the fungus *Trichoderma* and to test them against skin cancer protein. The metabolites were extracted in 80% methanol from the fungal biomass of *Trichoderma* isolated from mangrove sediment. The crude methanol extract was purified and analysed for the secondary metabolites by GC-MS. Three predominant compounds (heptadecanoic acid, 16 methyl-, methyl ester; 9,12-octadecadienoic acid; cis-9-octadecenoic acid) identified in the extracts were screened against the skin cancer protein (Hsp90) by *in-silico* docking method. Of the compounds, heptadecanoic acid, 16 methyl, methyl ester was the most potent having the docking score of −11.4592 Kcal/mol. This value was better than the standard drug “dyclonine”. This work recommends the heptadecanoic acid, 16 methyl, methyl ester for further *in vitro* and *in vivo* studies towards its development as anticancer drug.

## 1. Introduction 

 Marine environment is an excellent source of novel compounds with higher pharmaceutical applications [1, 2]. Marine-derived fungus produces a variety of metabolites having enormous biomedical applications. The low-molecular-weight secondary metabolites are produced by filamentous fungi, plants, algae, bacteria, and animals in response to environmental abiotic and biotic stress [3–5]. Among the fungi, metabolites produced by the *Trichoderma* species are known to have anticancer activity [6, 7]. Skin cancer is growing as a dreadful human disease as compared to other cancers [8]. However, potent drugs to cure skin cancer are increasingly necessary. Hence, the present study was undertaken to identify the predominant metabolites present in two species of *Trichoderma* species (*Hypocrea* spp.) isolated from mangrove sediment and also to test them against skin cancer protein (4,5-diarylisoxazole Hsp90 chaperone) using *in-silico* molecular docking methods.

## 2. Material and Methods

 Two strains of *Trichoderma* namely *Hypocrea lixii* TSK8 (JQ809340) and *Hypocrea estonica* SKS1 (JQ611722) were isolated from mangrove sediment by using *Trichoderma* selective medium [9] and stored at 4°C. The two strains were inoculated in a production medium with pH 7.2 and incubated at 28°C for 12 days and biomass was then harvested. 

### 2.1. Extraction of the Intra Cellular Secondary Metabolites

 The fungal biomass was extracted for intracellular metabolites [10] with some modifications. The fresh biomass was washed three times with sterile distilled water to remove adherent filtrate, and then blotted between folds of sterilized filter paper. The biomass was crushed in a mortar, using 80% methanol as solvent, and this extraction was repeated three times, and left in separating funnel for 15 min for precipitation. The crude extract was filtered through Whatman No.1 filter paper and the filtrate was dried under vacuum at 40°C.

### 2.2. GC-MS Analysis (Gas-Chromatography Mass Spectroscopy)

The filtrate was analysed for secondary metabolites by using GCMATE II GC-MS (Agilent). 1 *μ*L of the extract was injected through HP-5 capillary column, maintained at the temperature at 220°C and Helium as carrier gas. After analysis, the compounds were identified by matching with the known compound library.

### 2.3. Retrieval of Protein Structure

 The target 4,5 diarylisoxazole HSP90 chaperone protein (PDB ID: 2VCJ), having the resolution of 2.0 Å, was retrieved from the protein data bank (PDB) (http://www.rcsb.org/pdb/). A standard compound Dyclonine known to have good inhibitory potential against the same skin cancer protein was also docked to compare the effectiveness of the secondary metabolites. Structural and active site studies of the protein were done by using CASTP (Computed Atlas of Surface Topography of Proteins) and Pymol molecular visualization software.

### 2.4. Compounds Screened

 Three compounds, namely, Heptadecanoic acid, 16methyl-, methyl ester; 9,12-Octadecadienoic acid; and cis-9-Octadecenoic acid, identified by GC-MS analysis, were screened against the skin cancer protein. The compound details were retrieved from the Pubchem database and the chemical structures were generated from SMILES notation (simplified molecular input line entry specification) by using the Chemsketch Software (http://www.acdlabs.com).

### 2.5. Active Site Prediction

 Active site of the target protein was predicted by using “Active site prediction tool” from SCFBio Server (http://www.scfbio-iitd.res.in/dock/ActiveSite .jsp)  which requires a. pdb file as an input and this tool explains the total number of active sites along with information on their amino acid sequence, cavity points, and the average volume of the cavity.

### 2.6. Docking Methods 

 ArgusLab 4.0.1, most common and freely available software, was used for docking analysis. The inhibitor and target protein were geometrically optimized and “Argus dock” docking engine was used. Calculation type was set to “dock” mode whereas “flexible mode” was selected for the ligand. Grid resolution was set to 0.40 Å. Least energy represented the easy binding character of ligand and receptor.

### 2.7. Ligand Binding Sites Prediction

 After docking the docked structure was saved as “.pdb” file and further explored to predict the binding sites using “Ligand Explorer” software. The predicted binding sites, based on the binding energy, and amino acids make up the binding cavity. Here ligand binding site represents the site where the ligands most efficiently bind with the protein, among all the active sites.

### 2.8. Drug Likeliness Prediction

 Ligand property was predicted by using “Lipinski Drug Filters” (http://www .scfbio-iitd.res.in/utility/LipinskiFilters.jsp). Lipinski rule of five helps in distinguishing drug-like and non-drug-like properties and predicts high probability of success or failure due to drug likeliness for molecules. The Lipsinki filter helps in early preclinical assessment and thereby avoiding costly late-stage preclinical and clinical failures. 

## 3. Results and Discussion

**Figure 1:**
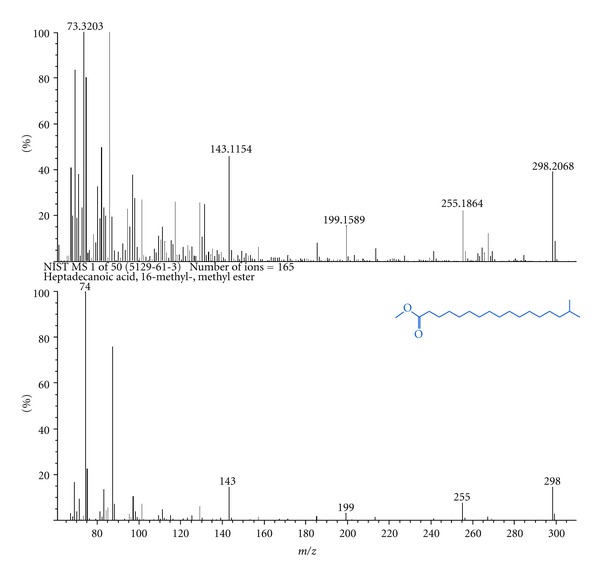
GC-MS result of potential compound Heptadecanoic acid, 16 methyl derived from *Hypocrea lixii* TSK8.

 The metabolites present in two fungal strains of *Hypocrea* species were analyzed in GC-MS and the compound (Heptadecanoic acid, 16 methyl) which showed the best result against skin cancer protein is shown in Figure 1. GC-MS result revealed the presence of fatty acids in both the strains. This could be attributed to the fact that polymerisation of acetate results in the formation of a fatty acid or a polyketide [11]. *Trichoderma *species are already known to produce fatty acids from mussels [11]. They also produce novel cytotoxic compounds such as trichodenone A, B, and C which exhibit significant cytotoxicity against leukemia P388 cell line [12, 13]. They have enormous pharmaceutical values such as antibacterial, antiviral [13], antiprotozoal [14], antifungal activities [15], and anti cancer [16].

 The protein Hsp90 is abundant in eukaryotic cells and its expression increases when cells are exposed to a variety of stresses [17]. Hsp90 contains three conserved domains: an N-terminal ATP-binding domain, a middle domain, and a carboxy-terminal domain [18]. Hsp90 is upregulated 10-fold in tumour cells suggesting that it helps maintaining tumour cell growth and/or survival. Another role for Hsp90 in the maintenance of tumour cells is its ability to inhibit apoptosis [19]. Inhibitors of the Hsp90 molecular chaperone are showing considerable promise as potential chemotherapeutic agents for cancer [20]. Hence, the present work tested the metabolites against 4,5-diarylisoxazole Hsp90 chaperone is a skin cancer protein.

**Figure 2:**
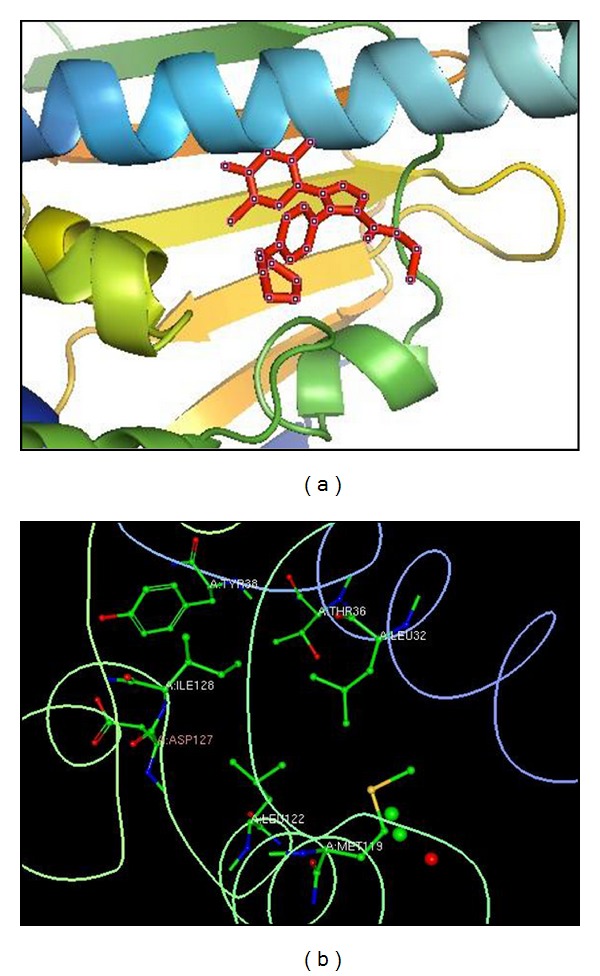
Molecular visualization of interaction between Heptadecanoic acid, 16 methyl-, methyl ester and the target protein. (a) Protein-ligand interaction (Pymol software). (b) Amino acids in the binding pocket (Coil: TYR38, THR36, ILE128, ASP127, MET119); (Alpha: LEU122, LEU32) (RCSB Ligand Explorer).

 Totally 47 active sites were predicted in the target protein by the “Active site prediction tool”. ArgusLab molecular docking software 4.0.1 was used to dock fatty acid compounds against the skin cancer protein (4,5-diarylisoxazole Hsp90 chaperone). The docking interaction of the protein and ligand, and the predicted ligand binding site residues are shown in Figures 2(a) and 2(b), respectively. The docked ligand molecules were selected based on docking energy and good interaction with the active site residues and the results are shown in Table 1. Of three compounds, Heptadecanoic acid, 16 methyl, methyl ester was the most potent having the least docking score of −11.4592 Kcal/moL. This value was better than that of the potent drug “Dyclonine” [21] which showed the docking score of −10.088. Lesser the docking score more is the binding capacity of the ligand. Hence, the present study suggested that heptadecanoic acid, 16 methyl, methyl ester could be considered for further *in vitro *and *in vivo* studies towards development of ant-skin-cancer drug.

## 4. Conclusion

 The present study showed that fatty acid derivative obtained from *trichoderma* species could be a potent inhibitor against skin cancer protein on the basis of docking scores. We anticipate that further exploration of the functions and molecular mechanisms of the compound will facilitate a better understanding for the control of skin cancer and in development of anticancer drugs.

## Figures and Tables

**Table 1:** Docking results of fungal compounds against skin cancer protein.